# Efficiency of naringin against reproductive toxicity and testicular damages induced by bisphenol A in rats

**DOI:** 10.22038/ijbms.2019.29757.7184

**Published:** 2019-03

**Authors:** Soheila Alboghobeish, Masoud Mahdavinia, Leila Zeidooni, Azin Samimi, Ali Akbar Oroojan, Saeid Alizadeh, Mohammad Amin Dehghani, Akram Ahangarpour, Layasadat Khorsandi

**Affiliations:** 1 Department of Pharmacology, School of Medicine, Student Research Committee of Ahvaz Jundishapur University of Medical Sciences, Ahvaz, Iran; 2 Department of Toxicology, School of Pharmacy, Jundishapur University of Medical Sciences, Ahvaz, Iran; 3 Department of Toxicology, School of Pharmacy, Student Research Committee of Ahvaz Jundishapur University of Medical Sciences, Ahvaz, Iran; 4 Department of Physiology, Student Research Committee of Ahvaz Jundishapur University of Medical Science, Ahvaz, Iran; 5 Health Research Institute, Diabetes Research Center, Department of Physiology, Ahvaz Jundishapur University of Medical Sciences, Ahvaz, Iran; 6 Department of Histology, School of Medicine, Ahvaz Jundishapur University of Medical Sciences, Ahvaz, Iran

**Keywords:** Bisphenol A, Endocrine-disrupting – chemicals, Naringin, Oxidative stress, Testicular toxicity

## Abstract

**Objective(s):**

Bisphenol A (BPA) as a synthetic compound is applied in many plastic industries. BPA has been reported to have endocrine-disrupting feature with cytotoxic effects. The study aimed to evaluate the efficiency of Naringin against testicular toxicity induced by BPA in adult rats.

**Materials and Methods:**

The animals were assigned into six groups of control, BPA-treated (50 mg/kg), BPA+Naringin-administrated (40, 80, 160 mg/kg) and Naringin-treated (160 mg/kg) for 30 days. At the end of experiments, testicular weight, total testicular protein, epididymal sperm count, testicular enzymes, serum follicle-stimulating hormone (FSH), luteinizing hormone (LH), testosterone and estradiol, testicular enzymatic and non-enzymatic antioxidants and histopathology of testis tissue were evaluated by their own methods.

**Results:**

The results showed a reduction in testicular weight, total testicular protein, epididymal sperm count, testicular enzymes (alkaline phosphatase and lactate dehydrogenase) and decrease in the serum TSH, LH, testosterone and estradiol in BPA-administrated rats. Furthermore, BPA reduced the enzyme activities of glutathione peroxidase, superoxide dismutase, and catalase in testis tissue. Also, BPA caused an induction in lipid peroxidation and increase in reactive oxygen species levels, whereas it decreased the glutathione content of testis tissue. Histological findings exhibited seminiferous tubules vacuoles, atrophy and separation of the germinal epithelium in BPA-administrated rats. Oral administration of Naringin along with BPA normalized the biochemical, morphological and histological changes and reduced the testicular toxic condition.

**Conclusion:**

These results demonstrated that Naringin significantly managed male reproductive toxicity by antioxidant capabilities, preventing morphological modifications and escalating defense mechanism, thereby reducing oxidative stress from BPA-induced damage.

## Introduction

Statistical surveys show that 72.4 million couples in the world suffer from infertility problems (1), and about 90% of male infertility is due to low sperm count, a decrease in sperm quality, or both. There are many factors contributing to sperm defects, such as environmental pollutants, genetic abnormalities, alcohol or cocaine use, smoking and hormone deficiency (2). Bisphenol A (2, 2-bis (4-hydroxyphenyl) propane; BPA) is one of the most abundant chemical produced around the world (about 6 billion pounds a year) (3). This material that is used to make polycarbonate plastics and epoxy resins contains two unsaturated phenolic rings and is considered to be an estrogenic endocrine-disrupting chemical. Polycarbonate plastics and epoxy resins are used as coatings for metal cans and various plastic products such as toys, water pipes, drinking bowls, glasses, sports equipment, dental monomers and medical equipment (4). It has been reported that BPA may release from the walls of containers (5) and penetrate into the blood circulation and exert the genotoxic and cytotoxic effects (6). Also, heating of plastic cans exacerbates the BPA release (7). Given the high availability and side effects of the BPA on infertility, it is possible that increased contact with the BPA may be the underlying cause of increased infertility and breast cancer in industrialized countries over the past 50 years (3, 8, 9). In addition, *in vitro* studies have shown that the BPA binds to the receptors of estrogen (10), androgen, and thyroid hormones as well as peroxisome proliferator-activated receptor gamma (11). Most studies on male rodents reported that the BPA reduces fertility factors. For example, exposure to the BPA has been reported to reduce sperm count (7, 12, 13), and sperm motility (14), and increase sperm DNA impairment in rats and mice (15-17). Furthermore, the BPA exposure caused a decrease in testosterone level in rats (15, 18) and mice (19). It is known that the BPA produces reactive oxygen species (ROS), such as hydroxyl radical, hydrogen peroxide, and superoxide anion in the body (20). In addition, the BPA can exert reproductive toxicity and damage spermatogenesis through production of ROS and impairment of natural antioxidant (21). The use of pharmaceutical properties of plants in the treatment of male reproductive toxicity is gaining more momentum due to its availability and affordability (22).

Naringin (NG) is a flavone found in citrus fruits, tomatoes, cherries, grapefruit, and cocoa (23). This compound acts as a nontoxic natural product with several functions, such as anticancer (24), anti-oxidative (25), anti-inflammatory (26), nephron-protective (27) and hepatoprotective activities (28). In one study, it was reported that NG restored normal testicular function, including sperm parameters in type 1 diabetic rats (29). With this background in mind, the present study aimed to assess the effect of BPA against the testicular function of Wistar rats and the therapeutic efficacy of NG in this regard. It is notable that the research was conducted using biochemical and histological methods.

## Materials and Methods

***Chemicals***

BPA and NG were purchased from Sigma-Aldrich Corp. (St. Louis, MO, USA). ). Bovine serum albumin (BSA), 2,7- di chloro fluorescein diacetate (DCFH-DA), 3,4 3-(4,5-dimethylthiazol-2-yl)-2, 5-diphenyltetrazolium bromide (MTT), thiobarbituric acid (TBA), trichloroacetic acid (TCA), 1,1,3,3-tetramethoxypropane, reduced glutathione, oxidized glutathione, and Coomassie Brilliant Blue powder were purchased from Sigma-Aldrich (St Louis, Missouri, USA). Sucrose 5, 5’-dithiobis-2-nitrobenzoic acid (DTNB), dimethyl sulfoxide (DMSO), NaCl, KCl, CaCl_2_, MgCl_2_ and NaHCO_3_ were obtained from Merck Company (Darmstadt, Germany). Glutathione peroxidase (GSH-Px; Cat No. S0058), and glutathione (GSH) assay kit (Cat. No. S0052) were purchased from Beyotime institute of Biotechnology (Jiangsu, China). 

***Study animals***

This experimental study was performed on 36 male Wistar rats aged 44-48 days weighing 160-180 g prepared from the Animal Center of Ahvaz Jundishapur University of Medical Sciences (AJUMS). The rats were tested in this study according to the guidelines of the Animal Ethics Committee (IR.AJUMS.REC.1395.141). They were kept at a temperature of 20 ± 4 ^°^C with a 12/12 hr light/dark cycle and free access to standard diet and water.

***Study test design***

The rats were divided into six groups with six rats in each group.

Group 1: The animals received 1 ml of olive oil emulsion for 30 days orally (control group).

Group 2: The animals received 1 ml of BPA (50 mg/kg body weight) in olive oil for 30 days orally.

Groups 3, 4 and 5: The animals received 1 ml of BPA (50 mg/kg body weight) in olive oil plus NG (40, 80 or 160 mg/kg body weight) for 30 days orally.

Group 6: The animals only received NG (at a concentration of 160 mg/kg body weight) for 30 days orally.

Overnight after the trial, the animals were sacrificed by cervical dislocation under mild anesthesia. Plasma samples were collected from the heart blood and centrifuged at 3500 rpm for 20 min and stored at -80 ^°^C until the hormonal testing. The right and left testicles of all animals were immediately dissected and the testicular weight and morphology (width, length, and volume) were examined in each group. The testicular volume was calculated using the following equation: 

Volume = (D2/4 × π) L × K (length [L], width [D], K = 0.9, π = 3.14) (30).

**Figure 1:**
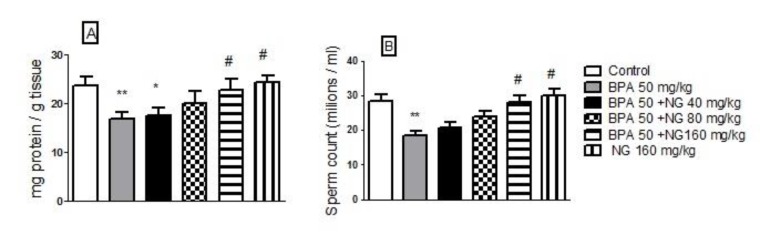
Effects of bisphenol A (BPA) and naringin (NG) on testis total protein (A) and sperm count (B). Each value was presented as means±SEM (n=6). *: Significantly different from control group (*P*<0.05), #: Significantly different from BPA-treated group (*P*<0.05), **: *P*<0.01. *P*-values were from one-way ANOVA, followed by Tukey’s test for multiple comparisons

**Figure 2:**
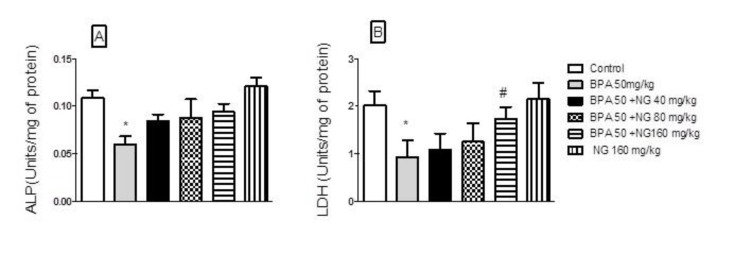
Effects of bisphenol A (BPA) and naringin (NG) on testicular homogenate enzymes: (A) Alkaline phosphatase (ALP), (B) lactate dehydrogenase (LDH). Each value was presented as means±SEM (n=6). *: Significantly different from control group (*P*<0.05), #: Significantly different from BPA-treated group (*P*<0.05). *P*-values were from one-way ANOVA, followed by Tukey’s test for multiple comparisons

**Figure 3:**
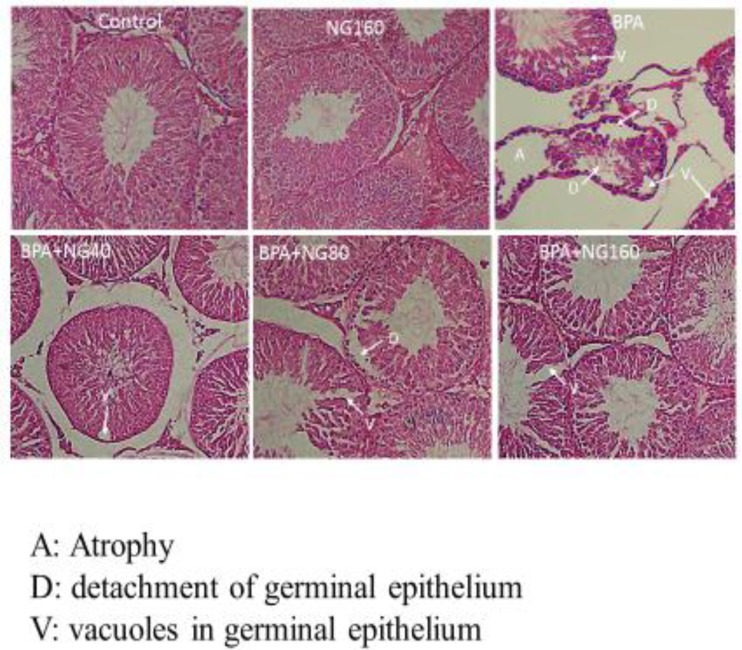
Effects of bisphenol A (BPA) and naringin (NG) on testicular histopathology (H&E, ×400). A: Atrophy, D: detachment of germinal epithelium, V: vacuoles in germinal epithelium

A: Atrophy; D: detachment; V: vacuoles in germinal epithelium

**Table 1:** Effect of Bisphenol A (BPA) and Naringin (NG) on final body weight, testis weight and testicular morphology (width, length and volume)

Parameters Treatment	Body weight (g)	Testis weight (g)	Testis length (mm)	Testis width (mm)	Testis volume (mm3)
Control	230.14±3.12	1.5±0.03	2.01±0.03	1.34±0.05	1.83±0.04
BPA 50 mg/kg	225.20±1.13	0.90±0.01*	1.85±0.1	1.07±0.15	1.368±0.12**
BPA 50 mg/kg + NG 40 mg/kg	224±5.7	1±0.03*	1.95±0.2	1.21±0.06	1.623±0.13#
BPA 50 mg/kg + NG 80 mg/kg	227±4.8	1.1±0.05	2±0.11	1.2±0.09	1.65±0.1#
BPA 50 mg/kg + NG 160 mg/kg	229.56±2.02	1.40±0.10#	2.08±0.09	1.2±0.07	1.716±0.08##
NG 160 mg/kg	230.44±4.30	1.5±0.22	2.12±0.07	1.3±0.07	1.895±0.07

Data are Mean ± SEM; n = 6. Difference between control and other groups is significant at *P* < 0.01

** () and *P* < 0.05 (). Difference between BPA-treated and other groups is significant at *P* < 0.01

## () and *P* < 0.05 (). *P*-values were from one-way ANOVA, followed by Tukey’s test for multiple comparisons.

**Table 2:** Effect of Bisphenol A (BPA) and Naringin (NG) on hormonal levels in serum of control and experimental animals

Parameters	Plasma FSH level (pg/ml)	Plasma LH level (pg/ml)	Plasma testosterone level (ng/ml)	Plasma estradiol level (ng/ml)
Treatment				
Control	33.30 ± 0.89	19.55 ± 2.10	3.98 ± 1.90	0.86 ± 0.07
BPA 50 mg/kg	12.42 ± 0.73**	9.18 ± 0.36**	1.07 ± 0.25*	0.58 ± 0.09*
BPA 50 mg/kg + NG 40 mg/kg	20.9±5.7##	10.76±0.03	1.95±0.2	0.68 ± 0.07
BPA 50 mg/kg + NG 80 mg/kg	19.75 ± 0.61##	15.27 ± 1.00##	2.04 ± 2.81	0.69± 0.04
BPA 50 mg/kg + NG160 mg/kg	21.95±2.02##	16.00±0.10##	3.08±0.09#	0.76± 0.06
NG 160 mg/kg	32.99 ± 1.24	19.09 ± 0.70	3.90 ± 3.03	0.83 ± 0.09

Data are Mean ± SEM; n = 6. Difference between control and other groups is significant at, *P* < 0.01

** () and *P* < 0.05 (). Difference between BPA-treated and other groups is significant at *P* < 0.01

## () and *P* < 0.05 (). FSH: follicle stimulating hormone; LH: luteinizing hormone. *P*-values were from one-way ANOVA, followed by Tukey’s test for multiple comparisons.

**Table 3:** Effect of Bisphenol A (BPA) and Naringin (NG) on enzymatic and non-enzymatic antioxidants

Groups	Control	BPA 50 mg/kg	BPA 50 mg/kg +NG 40 mg/kg	BPA 50 mg/kg +NG 80 mg/kg	BPA 50 mg/kg +NG 160 mg/kg	NG 160 mg/kg
Variables						
MDA (nmol/mg protein)	6.65± 2.3	3.31± 0.7*	3.93± 0.4	4.83± 0.6	5.1±0.88#	6.6±0.81
ROS (% of Control)	100 ±10.1	197.87±12.61***	166.65±11.76	141.76±11,6##	117.64±14.7###	94.89±17.1
GSH (µg/ mg of protein)	0.7±0.09	0.2±0.01***	0.29±0.02	0.42± 0.03	0.58±0.02##	0.8±0.01
Catalase (µmol/ mg of protein)	60.22±1.34	31.58±2.31*	42.3±3.8	53.7±2.9	53.90±1.72	61.28±1.87
SOD (U/mg of protein)	2.5±0.56	1.3±0.99*	1.54±0.29	1.72±0.59	2.02±0.48#	2.87±0.19
GSH-Px (µmol/ mg of protein)	2.3±0.47	1.2±0.17*	1.3±0.05	1.43±0.07	1.7±0.45#	2.4±0.99
MMP (% of Control)	100 ±16.7	64.87±12.61**	73.65±11.76	85.76±11,6#	95.64±14.7##	99.99±17.1

*P*-values were from one-way ANOVA, followed by Tukey’s test for multiple comparisons.

*** Data are Mean ± SEM; n = 6. Difference between control and other groups is significant at *P* <0.001(),*P* < 0.01 () and *P* < 0.05 ().

### Difference between BPA-treated and other groups is significant at *P* <0.001(), *P* < 0.01 () and *P* < 0.05 (). MDA: Malondialdehyde, ROS: Reactive oxygen species, GSH: Glutathione, SOD: Superoxide dismutase, CAT: Catalase, GSH-Px: Glutathione peroxidase, and MMP: Mitochondrial membrane potential.

**Table 4:** Effect of Bisphenol A (BPA) and Naringin (NG) on tubular diameter and epithelial height

Treatment	Seminiferous tubule diameter (μm)	Epithelial height (μm))
Control	223.7±9.52	85.4±2.85
BPA 50 mg/kg	136.65±13.13***	56.92±0.61***
BPA 50 mg/kg + NG 40 mg/kg	139.65±5.7***#	49.58±1.33***#
BPA 50 mg/kg + NG 80 mg/kg	147.52±4.8**##	64.53±2.5**##
BPA 50 mg/kg + NG160 mg/kg	206.56±12.02*###	83.35±3.10###
NG160 mg/kg	219.59±4.30	86.64±2.22

*P*-values were from one-way ANOVA, followed by Tukey’s test for multiple comparisons.

*** Data are Mean ± SEM; n = 6. Difference between control and other groups is significant at *P* <0.001(), *P* < 0.01 () and *P* < 0.05 ().

### Difference between BPA-treated and other groups is significant at *P* <0.001(), *P* < 0.01 () and *P* < 0.05 ().

***Rat sperm count***

Epididymis cauda was separated from the testicles of each rat, and after conversion into small pieces, 3 ml of normal saline was added. For sperm count, one drop of the above solution was transferred on a Neubauer chamber (depth 0.100 mm and area 0.0025 mm^2^; HBG Henneberg-Sander GmbH, Gießen, Germany) and sperm count was performed manually using an optical microscope (Olympus Light Microscope; Olympus Corp, Japan). At the end, the data were calculated as the sperm count per ml (31). 

***Sample collection***

In this stage, 0.1 mol/liter hydrochloric acid buffer (pH = 7.4) was added to the right testis and mixed with a homogenizer and centrifuged at 3000 rpm for 15 min.

***Estimation of total testicular proteins***

The total testicular proteins were determined using the Lowry method (32) and using BSA (1 mg/ml) as the standard. The absorbance was read by spectrophotometer at a wavelength of 750 nm.

***Determination of alkaline phosphatase and lactate dehydrogenase enzymes in rat testicles***

Diagnostic kits using spectrophotometer were applied to determine the alkaline phosphatase (ALP) and lactate dehydrogenase (LDH) enzymes in testicular homogenization.

***Measurement of the ROS level in the rat testicles***

The ROS level in testicular tissue was measured using DCFDA, which was converted to DCF fluorescence by cell peroxides. In a nutshell, 2 ml of testicular homogenization was mixed with 4 ml of DCFDA in 1.25 mM of methanol and incubated at 37 ^°^C for 15 min. Fluorescence level was measured using a fluorimeter (24, 33).

***Measurement of glutathione in rat testicles***

The GSH levels were measured using the method of Thomas and Screnska. Thus, 1 ml of testicular homogenization was incubated with 1 ml of 20% TCA and 1 ml of EDTA for 5 min and then centrifuged at 1000 rpm in 4°C for 30 min. Next, 200 μl of supernatant was mixed with 1.8 ml of DTNB. GSH reacts with DTNB and forms a yellow compound. The absorbance was read at a wavelength of 412 nm (34).

***Measurement of malondialdehyde level in rat testicles***

The level of lipid peroxidation was determined by measuring malondialdehyde (MDA). Thus, 1 ml of testicular homogenization was heated with 1 ml of 20% TCA and 2 ml of 0.67% TBA for 1 hr in boiling water. After cooling down, the mixture was centrifuged and the absorbance of supernatant was read at a wavelength of 532 nm. The MDA level was calculated using a molar coefficient of è = 1.56 × 105/M/cm (35).

***Measurement of catalase in rat testicles***

The catalase was measured using Goth method. Thus, 500 μl of Tris-HCl, 1 ml of H_2_O_2_ and 50 μl of the sample were mixed and incubated for 10 min and then 500 μl of ammonium molybdate 4-hydrate was added and the absorbance was read at a wavelength of 410 nm (36).

***Measurement of*** ***Superoxide dismutase*** ***level in rat testicles***

The superoxide dismutase (SOD) activity was measured using a xanthine/xanthine oxidase system to generate superoxide radical. Optical density was determined using spectrophotometer (UV- 1601, Shimadzu) at a wavelength of 550 nm (37). The SOD activity was expressed as a unit per milligram of protein (U /mg protein).

***Measurement of glutathione peroxidase activity***

The GSH-Px activity was calculated by measuring the oxidation level of reduced GSH to oxidized GSH via H_2_O_2_ catalyzed by GSH-Px. A laboratory kit was used for this purpose (Jiancheng Bioengineering, China) (38).

***Measurement of hormones***

ELISA kits were used to measure the hormones of follicle-stimulating hormone (FSH), luteinizing hormone (LH), estradiol and testosterone (DRG Instruments GmbH, Marburg, Germany). 

***Histopathological examination of rat*** ***testicles***

After blood sampling, the left testicles of the animals were separated and placed in the Bouin’s fluid for histopathological examination. After overnight, the samples were cleaned by alcohol dehydration in benzene and subjected to paraffin wax. Sections of 5 to 7 micrometers were prepared from the tissue and stained with hematoxylin/eosin staining method. Six microscopic slides were explored to evaluate tissue changes in each rat, and their average was calculated. The diameters of tubules and lumen were measured using Motic Images plus 2.0 image analysis software (Motic, Hong Kong, China). The height of the epithelium was calculated by removing the diameter of the lumen from the diameter of the tubules. For each animal, 150 tubules were examined (37, 39).

***Statistical analysis***

All results were expressed as mean ± SEM using Graph Pad Prism software (version 5.04) and then analyzed by one-way ANOVA and then by Tukey’s test. Kruskal-Wallis and then Mann-Whitney U tests were used for data with non-normal distribution and non-homogeneous variance. *P*<0.05 was considered as statistically significance level.

## Results

***Effect of BPA and NG on body weight, testis weight and testicular morphology (width, length and volume)***

According to the results, there was no difference in body weight between the research groups. In addition, the weight of testes significantly decreased after BPA treatment, in comparison with normal control rats. Furthermore, an increase in testis weight was indicated in the group treated with NG plus BPA (*P*<0.05). On the other hand, an insignificant difference was observed between the NG and control rats (*P*>0.05). The morphological findings of the rat testicles represented a decrease in testicular volume after BPA treatment (*P*>0.05), and a significant increase was observed in the volume after administration of NG plus BPA (*P*<0.05)(Table 1).

***Effect of BPA and NG on total testicular protein***

By using BPA, total testicular protein was significantly reduced compared to normal rats (*P*<0.01). In contrast, in rats that used NG plus BPA, there was a significant increase in total testicular protein content compared to rats that used only BPA (*P<*0.05) (Figure 1A).

***Effect of BPA and NG on sperm count***

The results showed that the sperm count was decreased significantly with the use of BPA compared to the control group (*P<*0.05), but after NG administration, it was close to normal compared to BPA-treated group (*P<*0.05); however, no significant difference was found between the NG and the control groups (Figure 1B).

***Effect of BPA and NG on testicular enzymes***

Results demonstrated that the levels of ALP and LDH were significantly decreased in testis (*P<* 0.05) after BPA treatment. Furthermore, an increase (*P<*0.05) in ALP and LDH of testis was observed after the concomitant administration of NG plus BPA (Figure 2A, B).

***Effect of BPA and NG on plasma levels of LH, FSH, testosterone and estradiol***

The amount of FSH, LH, estradiol, and testosterone in plasma were significantly reduced due to BPA-induced toxicity (respectively, *P*<0.05, 0.05, 0.01), but after treatment with NG, there was a growth in the amount of these hormones. Furthermore, no change was observed in this parameter in animals with independent administration with only NG (Table 2).

***Effects on enzymatic and non-enzymatic antioxidants***

After BPA intoxication, MDA and ROS amounts were significantly increased (*P<*0.05, 0.001), but their levels significantly decreased when NG was administered to BPA-intoxicated rats (respectively, *P<*0.05, 0.001). In addition we showed an induction in the GPx enzyme after BPA intoxication (*P<*0.05); however, this factor was elevated in NG-BPA co-treatment groups. The GSH level was reduced following the BPA intoxication, but incremented after NG-BPA intake (*P<* 0.01). On the other hand, SOD and CAT levels were significantly decreased after BPA intoxication (*P<*0.05), and increased after treatment with NG (*P<*0.05) (Table 3).

***Effects on testicular histopathology***

The seminiferous tubule diameter and epithelial height were decreased in BPA-treated rats, compared to the control group (*P<*0.001). But co-administration of NG (80, and 160 mg/kg) with BPA significantly increased the tubular diameter, and epithelial height (respectively, *P<*0.05, 0.01, 0.001) (Figure 3 and Table 4). In the control and NG 160 mg/kg groups, testicular tissues appeared normal. In the BPA-treated group, atrophy and separation of the germinal epithelium was observed in the most of the seminiferous tubules vacuoles. In group BPA+NG 40 mg/kg, the separation was observed in some of the tubes vacuoles. In group BPA+NG 80 mg/kg, the testicular tissue damage was less than that of the BPA+NG 40 group. There was no separation in group NG 160 mg/kg and only some tubes had vacuoles.

## Discussion

Increased toxic effects of drugs and environmental substances have been recently reported on reproductive system (40). Plants and their bioactive components are the most extensively accessible materials that have the ability to scavenge free radical ions (41). Therefore, this context investigated the possible effects of NG treatment against BPA-induced reproductive toxicity and testicular damage in adult male rats. The significant decrease in testicular mass and volume may be due to reduced bioavailability of gender hormones, which points to the reproductive endocrine stipulation of the male. Testis weight and volume are directly related to the mass of the spermatogenic cells. Therefore, reduced testis mass and volume affects spermatogenesis activity with decreased germ cells in turn. In addition, it was noted that BPA intoxication decreases the testis weight and volume of the experimental rats by damaging the important molecules, such as proteins in the testis. These results were in line with studies reporting the testicular weight loss because of exposure to BPA (42). Furthermore, we showed an increase in the testes weight and volume in NG-BPA co-treated animals due to therapeutic efficacy and protective potential of NG.

In addition, we showed a reduction in amount of testicular total protein of BPA-treated rats. Previous studies have suggested that environmental testicular toxicants directly exert antagonistic effects on proteins and lipids of reproductive organs (43). Multiple stimulatory and inhibitory factors are in testicular fluid that selectively change the production of proteins (18). As a result, alteration in testicular protein level proposed a decrease in the synthetic activity of testes. In addition, we showed that NG can somewhat reduce the testicular total protein level.

The epididymal sperm count is among the most sensitive tests used to evaluate the spermatogenesis due to providing the result of all stages of meiosis, spermatogenesis, and transition in the epididymis. It is suggested that a reduction in sperm count is related to male infertility (44). In our study, NG treatment increased the sperm count, which might be due to suppression of the disturbances induced by free radicals in sperm.

LH, FSH and testosterone are essential hormones for spermatogenesis. The LH hormone is secreted from the pituitary gland and triggers the testosterone production. On the other hand, there is a need for testosterone to produce and maintain sperm (45). In addition, FSH and testosterone stimulate spermatid growth and sperm release (46). In our study, the BPA administration reduced the levels of LH, FSH and testosterone, possibly due to effects on Sertoli and Leydig cells. The results of our study were consistent with the findings of previous studies, in which the BPA caused a change in the hypothalamic-pituitary-gonadal axis and decreased sertoli cell function (47). In addition, previous studies suggested that reducing testosterone in rats treated with BPA may be due to interference with the proliferation and function of Leydig cells (48). According to Akingbemi *et al.* (49), the BPA has an effect on the pituitary and disrupts testicular activity, thereby reducing the LH release. The BPA has also been reported to reduce the testosterone production from Leydig cells by reducing the activity of 17α-hydroxylase or increasing aromatase activity (50).

However, the plasma levels of LH, FSH and testosterone were higher in animals treated with NG-BPA than in animals treated with BPA, which is consistent with increased spermatogenesis in the NG-treated group. This effect may be due to the direct or indirect effect of NG on the oxidative damage of cells. These results were consistent with previous reports in which NG treatment increased testosterone levels in diabetic rats (29). According to our results, estradiol level is reduced in BPA-exposed groups. Previous studies reported that low BPA doses have estrogen-inducing activity, while high BPA doses inhibit estrogen production (51). In addition, our results showed that NG was able to normalize the effects of changes caused by BPA on the estradiol levels.

Marker enzymes are sensitive indicators that are released when impairment happens in a tissue. It is an indicator used to evaluate the management of diseases. Most of the particular enzymes, such as LDH and ALP, are affected when there is an impact on homeostasis in the biological system. LDH is a cytoplasmic bi-directional enzyme capable of forming pyruvate and lactate in cells. In addition, LDH extensively exists in Sertoli and spermatogenic cells, which is importantly involved in testis energy generation, biotransformation and translocation of hydrogen from the cytoplasm to mitochondria by redox coupling α-hydroxyl acid/α-keto acid associated with spermatozoa metabolism (43). The LDH enzyme in testicles is associated with the maturation of germinal epithelial layer of seminiferous tubules and postmeiotic spermatogenic cells (18, 52). BPA-treated animals indicated a significant induction in LDH activity. In contrary, the administration of NG enhanced the activity of this enzyme. The testicular levels of ALP enzyme in rats declined after BPA administration, but increased by NG administration. The ALP enzyme in testicles is mainly of testicular and epididymal origin (53). In our study, reduced ALP activity revealed that the treatment with BPA may decrease the activity of testicular tissues.

Free radicals and lipid peroxidation are two leading factors in testicular pathology. The lipid peroxidation occurs in unsaturated lipids and is involved in the formation of active oxygen (54). On the other hand, the sperms are subject to increased lipid peroxidation because of the prevalence of unsaturated fatty acids in their membranes (55). In the present study, there was a significant increase in the incidence of testicular lipid peroxidation, probably due to the effect of BPA-induced oxidative damage. In this regard, previous study marked an induction in ROS generation due to the association of lipid peroxidation with abnormalities and decreased sperm counts (56). Spermatozoa are generally susceptible to injury, which is attributed to the deliberation of unsaturated fatty acids and also the ability of spermatozoa to generate ROS (57). BPA-motivated oxidative stress may involve in the destruction of intracellular ATP production, which directly displays the effect on declined sperm motility, improved cell penetrability, inactivation of biological enzymes and manufacturing of spermicidal products (58).

In contrast, the effects of NG may be due to its protective effects on increased activity of lipid peroxidation or the production of free radicals. NG can protect the cell from oxidative damage by inhibiting intracellular ROS production and eliminating the produced ROS (23). To confirm this, previous studies have shown the protective role of NG against ROS-induced cytotoxicity by using arsenic (59), carbon tetrachloride (60), lead (61), cadmium (62), or cisplatin (63) in rats. It has been suggested that the NG may be coupled with Cu and Fe ions through the 5-hydroxy and 4-carbon groups in their C ring, thereby reducing the ROS (64). The NG has also been reported to stimulate expression of many antioxidant-related genes and inhibit the activity of ROS-forming enzymes such as NADPH oxidase (65).

Degradation and eventual rupture of the cellular membrane that leads to the release of cell organelle substances may be due to lipid peroxidation (66). In addition, it was reported that the BPA might be associated with the damage in mitochondrial membrane (67). Thereby, NG displayed anti-lipid peroxidation activity and protective effect on cell membrane against ROS by its free radical scavenging property.

The oxidative stress is an imbalance between the amount of free radicals production and the presence of antioxidants. The antioxidant defense system contains molecular antioxidants, antioxidant enzymes and metallic chemical agents (68). Antioxidant enzymes (e.g., SOD, CAT and GPx) protect the living system from the destructive effects of ROS and reduce their oxidative damage to testicular cell membranes (69). Reducing SOD activity results in the accumulation of superoxide radicals, which in turn inhibits the CAT enzyme (70). Reducing CAT activity decreases the ability of the testicles to eliminate H_2_O_2_ produced after exposure to BPA. The H_2_O_2_ can cause rapid oxidative damage to lipids, proteins and DNA (71). In addition, the GPx may act directly as an antioxidant enzyme, which is involved in inhibition of sperm lipid peroxides (72) and H_2_O_2 _(73). The low GPx level in BPA-exposed rats can lead to an increase in H_2_O_2_ production or a decrease in GSH concentration (18). In the present study, the antioxidant enzymes (such as SOD, CAT and GSH-Px) were reduced in BPA-exposed animals, which is consistent with the results of Chitra *et al.* study (74).

The GSH plays an important role in cellular function, including H_2_O_2_ destruction, lipid peroxidation, and the amino acid translocation in the cell membrane (75). In the present study, reduced testicular GSH level in BPA-exposed rats may be responsible for increasing ROS in the testes. In addition, reduced activity of antioxidant enzymes such as CAT, GPx, and SOD highlights the harmful effects of BPA. NG, on the other hand, reduces ROS-induced oxidative stress by stopping the lipid peroxidation in the testicle and increasing the enzymatic and non-enzymatic antioxidants.

According to the histological results, a decrease was observed in the seminiferous tubules diameter and the epithelial height in the BPA-administrated rats, which is in agreement with Tamilselvan *et al.* study (8). In contrast, treatment with NG shifted this change towards normal status, probably due to the medicinal property of this glycoside. The results of the current research showed several vacuoles in the seminiferous tubule epithelium in the BPA-treated group. Meanwhile, there was a reduction in the count and size of these vacuoles in the BPA+NG-administrated group. In the BPA-treated group, atrophy and separation of the germinal epithelium was observed. On the contrary, the tissue was significantly protected against BPA-induced histopathological changes by NG administration.

## Conclusion

Based on the findings from the present study, the BPA exposure resulted in structural and functional impairments in rat testes and epididymis, leading to reproductive toxicity, spermatogenesis impairment and disturbing of the hormonal balance. However, administration of NG reduced the BPA-induced reproductive toxicity through modulating the lipid peroxidation level, decreasing the free radicals and maintaining homeostasis, emphasizing the potential therapeutic role of NG against BPA-induced reproductive toxicity.
